# Electro-Elastic Modeling of Thermal Spin Transition in Diluted Spin-Crossover Single Crystals

**DOI:** 10.3390/ijms232213854

**Published:** 2022-11-10

**Authors:** Karim Affes, Yogendra Singh, Kamel Boukheddaden

**Affiliations:** 1 GEMaC, CNRS, Université de Versailles Saint Quentin en Yvelines, 45 Avenue des Etats Unis, CEDEX, 78035 Versailles, France; 2 Laboratoire des Matériaux Multifonctionnels et Applications, Faculté des Sciences de Sfax, Université de Sfax, Route de la Soukra km 3.5, Sfax 3000, Tunisia

**Keywords:** spin transition, electro-elastic modelling, Monte Carlo simulations, diluted molecular materials, thermal hysteresis, spatiotemporal properties

## Abstract

Spin-crossover solids have been studied for many years for their promising applications as optical switches and reversible high-density memories for information storage. This study reports the effect of random metal dilution on the thermal and structural properties of a spin-crossover single crystal. The analysis is performed on a 2D rectangular lattice using an electro-elastic model. The lattice is made of sites that can switch thermally between the low-spin and high-spin states, accompanied by local volume changes. The model is solved by Monte Carlo simulations, running on the spin states and atomic positions of this compressible 2D lattice. A detailed analysis of metal dilution on the magneto-structural properties allows us to address the following issues: (i) at low dilution rates, the transition is of the first order; (ii) increasing the concentration of dopant results in a decrease in cooperativity and leads to gradual transformations above a threshold concentration, while incomplete spin transitions are obtained for big dopant sizes. The effects of the metal dilution on the spatiotemporal aspects of the spin transition along the thermal transition and on the low-temperature relaxation of the photo-induced metastable high-spin states are also studied. Significant changes in the organization of the spin states are observed for the thermal transition, where the single-domain nucleation caused by the long-range elastic interactions is replaced by a multi-droplet nucleation. As to the issue of the relaxation curves: their shape transforms from a sigmoidal shape, characteristic of strong cooperative systems, into stretched exponentials for high dilution rates, which is the signature of a disordered system.

## 1. Introduction

One of the most intriguing examples of molecular bistability is the ability of some metal complexes to switch between low-spin (LS) and high-spin (HS) states. Among these functional materials, the spin-crossover (SCO) molecular solids are excellent candidates for reversible memory devices [1,2,3], sensors of temperature, pressure, and gases [4]. They can also serve as switches of light emission in the electroluminescent devices containing spin-crossover (SCO) complexes [5,6], or to enhance plasmonic resonances [7].

The case of iron(II)-based SCO materials, with 3d^6^ configuration, is the most common in the literature. In these ferrous SCO compounds, the five d-orbitals of iron(II) are then split into three bonding, $t_{2g}$ and two anti-bonding, $e_{g}$, orbitals. When the strength of the ligand field, $\Delta =E\left(t_{2g}\right)-E\left(e_{g}\right)$, is much stronger (resp. weaker) than the electrons’ pairing energy, the metal ion can be in the LS $t_{2g}^{6}e_{g}^{0}\;$diamagnetic (resp. HS $t_{2g}^{4}e_{g}^{2}\;$paramagnetic) state with a total spin S=0 (resp. S=2). In Fe-based SCO materials, the spin transition between LS and HS is accompanied by an increase of ~10% of the Fe-ligand bond lengths due to the weakening of the metal–ligand bonds. This causes the changes in local volume of ~3–5% [8,9], as well as ligand metal–ligand bond angles by 0.5–80°, affecting their magnetic, vibrational and optical [10] properties. Nowadays, a large panel of experimental techniques is used to detect the spin transition. Among them, one can cite magnetic [11,12,13] and optical [14,15], Mössbauer spectroscopy [16], optical microscopy (OM) [17,18,19,20,21,22,23], X-ray diffraction [24], differential scanning calorimetry [25], diffuse reflectivity [26], etc. OM, in particular, allowed to investigate the spatiotemporal features of the spin transition on a unique single crystal. This enabled the imaging of the real-time transformation of the crystals along the thermal spin transition, with remarkable spatial (~0.3 µm) and temporal (up to 0.01 s) resolutions. External stimuli, such as pressure [5], temperature [27], electric field [28], magnetic field [29] and light [30], can also cause significant effects on the spin transition mechanism.

In crystalline state, the thermal properties of SCO materials can lead to a wide range of different thermal behaviors. For example, we mention the case of (i) first-order thermal spin transition (cooperative behavior), (ii) gradual spin transition [31] and (iii) multistep or incomplete spin transition [32]. In the latter case, a spatial self-organization of the spin states [33,34] is sometimes observed in the plateau region.

The elastic interactions between the SCO molecules are recognized as an essential ingredient in the origin of their cooperative character. The interactions between the SCO units are mainly caused by the volume differences between HS and LS molecules. If this volume difference is strong enough and the SCO molecules are well linked, then thermally induced first-order spin transitions take place. The cooperative nature of the solid results from combined short- and long-range interactions. Here, the short-range interactions relate to the shape and the distance between neighboring molecules. In contrast, the long-range interactions are caused by the local volume changes, which delocalize in the whole crystal through acoustic phonons [35,36,37].

Understanding the source of the interactions is critical, then, to constructing SCO materials with a high degree of cooperativity. The microscopic mechanism of interaction has been extensively studied, and various theoretical models [38] have been proposed to explain the experimental findings in a contextually sound manner. Among these models, we quote for example, (i) the Ising-like model [39], also used to describe SCO nanoparticles, (ii) the atom–phonon model [40,41], and the (iii) elastic models [42,43,44,45,46,47,48,49,50,51,52,53,54], which have been recently introduced. While the Ising models suffer from their qualitative nature and a lack of structural information, the elastic model accounting for the volume change at the transition appears much more realistic and has attracted a lot of interest these last years.

The study of metal ion dilution in solids is a typical experimental approach for elucidating the function of these elastic interactions. The mechanism and the range of the interactions between spin crossover units may be then studied by diluting the spin crossover metal centers. Within this technique, it is possible to regulate both the number of interconnecting centers and the intensity of those interconnections. The SCO molecules become increasingly isolated as they are randomly substituted by non-active impurities, disrupting the lattice cooperativity leading to more gradual transitions.

Previous experimental studies of metal dilution on SCO solids [55] have been performed on two-step SCO compounds [56] as well as on one-step SCO compounds, including hysteretic ones [57,58,59,60,61]. Diluting SCO complexes with isostructural but magnetically silent impurities has led to considerable information on the link between intermolecular contacts and the degree of cooperativity in the solid state. For example, the mixed crystal series $[{\mathrm{Fe}}_{x}{\mathrm{Zn}}_{1-x}\left(2-{\mathrm{pic}}_{3}\right]{\mathrm{Cl}}_{2}\mathrm{EtOH}\;$(x=0.007 to 1; 2-pic = 2-picolylamine) was the first series for which this approach was introduced [62], and the SCO curve first had a sharp step but grew more gradual as the Zn/Fe ratio rose. It also shows, in this material, that the dilution process energetically favors the HS state by lowering the transition temperature. Another study [63] reporting on the effects of zinc dilution concerned the mononuclear two-step spin crossover compounds $[{\mathrm{Fe}}_{x}{\mathrm{Zn}}_{1-x}\left(\mathrm{bapbpy}\right){\left(\mathrm{NCS}\right)}_{2}$ $\left(\mathrm{bapbpy}=N_{6},{\;N}_{6}^{\prime }-\mathrm{di}\left(\mathrm{pyridin}-2-\mathrm{yl}\right)-2,2^{\prime }-\mathrm{bipyridine}-6-6^{\prime }-\mathrm{diamine}\right)$. It demonstrated that increasing the zinc concentration results in the vanishing of the cooperativity at the threshold value x=0.24, with a decrease in the transition temperature. The role of cooperative elastic interactions between the SCO metal centers were validated by these findings, but not yet clearly explained. Other dilution investigations have shown similar results [62,64,65].

The dopant ion size profoundly affects the SCO characteristics. One of the most important reasons for this is that ions with a size larger than that of the HS Fe(II) cause a negative internal pressure in the LS lattice, thus favoring the HS state. When diluting Fe-based SCO compounds, alternative metal cations (Ni^2+^, Co^2+^, or Mn^2+^), that are not magnetically quiet, are also used to investigate the effects of different-sized metal ions [64,66]. Some of them, like Ni, have a neutral role because their size is located between that of Fe(II)-HS and Fe(II)-LS [55,61,65].

Another aspect of the metal dilution relates to its effect on the relaxation properties of the HS photo-induced metastable state. In this context, it is important to mention that A. Hauser and co-authors who discovered the LIESST (light-induced excited spin state trapping) effect [67] have also been the precursors in dynamical studies of diluted SCO solids. The influence of metal dilution on the light conversion [58,68,69,70,71,72,73] was studied for both excitation and relaxation processes. When iron(II) ions are diluted with a non SCO-active dopant, the HS-LS relaxation behavior changes from a self-accelerated to an exponential or stretched process owing to the decrease in cooperativity and to the inhomogeneous character of the lattice. On the other hand, from the thermodynamic point of view, the spin equilibrium temperature, $T_{eq}$, defined as the temperature for which $G_{HS}-G_{LS}=0$, where G is Gibbs free energy, was shown to be significantly influenced by metal dilution. Correlatively, for hysteretic spin transitions, the hysteresis loop’s width declined and eventually vanished with increasing dilutions.

The additional insight gained through these studies will be a fundamental step toward the explanation of the effect of dilution on the structural and thermal properties of the SCO materials. The primary goal of the present study is then to investigate the theoretical consequences of metal dilution on SCO systems. The investigations are made using the electro-elastic model, which has the merit of combining both electronic and elastic degrees of freedom of SCO phenomena. This model is extended here to include the effect of contaminants on SCO complexes, in which the interactions are modeled using springs.

The manuscript is organized as follows: Section 2 presents an extended version electro-elastic model adapted for diluted SCO materials and describes the simulation procedure. Section 3 reports the effects of dilution on the thermal spin transition and low-temperature relaxation of photo-induced metastable HS fractions. The effect of the dilution on the spatiotemporal aspects of the spin transition is also discussed. In Section 4, we conclude and outline the possible developments of the present work.

## 2. The Model and Simulations Procedure

We studied a distortable 2D square lattice with a fixed topology and open boundary conditions. The crystal is constituted by SCO units and dopants, also hereafter called defects. Each lattice site is associated with a fictitious spin, $S_{i}\;$, whose values are $S_{i}=-1$, for SCO site in LS state, $S_{i}=+1$ for a SCO site in HS and $S_{i}=0$ for a site occupied by a dopant (defect).

The total Hamiltonian of the system, taking into account for the spin states and lattice positions, is written:

(1) $$H=\underset{i}{\sum }\left(\Delta -k_{B}Tlng\right)S_{i}+A\underset{\left(i,j\right)}{\sum }(r_{ij}-R\left(S_{i},S_{j}\right))²+B\underset{\left(i,k\right)}{\sum }{\left(r_{ik}-R^{\prime }\left(S_{i},S_{k}\right)\right)}^{2}$$

The first term of the Hamiltonian (1) contains the energetic contribution $\left(\Delta =E_{HS}-E_{LS}\right)$ arising from the difference in the ligand–field energy between the HS and LS states at 0 K. The term, $k_{B}Tlng$, results from the entropic contribution $\;(k_{B}lng$) originating from the electronic, intramolecular vibrations and phonons changes between the LS and HS states. This degeneracy ratio, $g=\frac{g_{HS}}{g_{LS}}$, between the two states is assumed here, for simplicity, as temperature-independent and must be seen as an effective degeneracy. For the dopant, which does not show any spin transition, this energy is zero.

**Figure 1:**
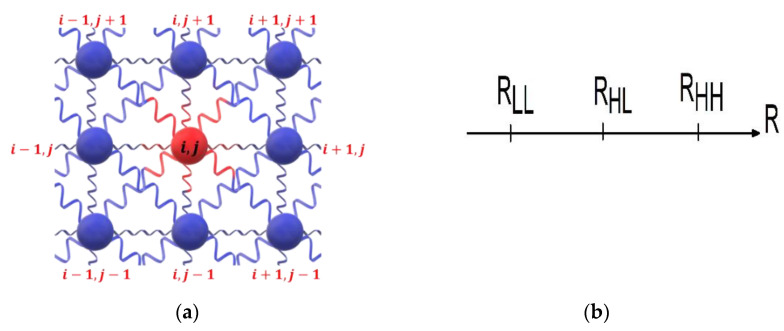
(**a**) Schematic representation of the configuration of the nearest (nn) and next-nearest (nnn) neighbor elastic interactions, considered in the present two-dimensional square lattice model. The red central site, labelled (*i*,*j*), is surrounded by four nn and four nnn sites (dark blue sites). The connections between the sites represent the springs. Each site can be in LS, HS or a dopant site. (**b**) Schematic view of the equilibrium nn bond lengths between two SCO sites. *R_LL_*, *R_HL_* and *R_HH_* are the equilibrium distances between LS-LS, HS-LS and HS-HS units, respectively.

The second and third contributions in (1) are the elastic interactions between nearest neighbors (nn) and next-nearest neighbors (nnn) sites, respectively, whatever their nature (SCO or defect). The parameters, A and B, are the corresponding nn and nnn elastic constants, assumed here as independent from the nature (SCO, defect) of the connected sites. The quantity, $r_{ij}=||{\vec{r}}_{i}-{\vec{r}}_{j}||$ (resp. $r_{ik}$) is the instantaneous distance between the nn (resp. nnn) nodes i and j (resp. i and k). In the expression (1), the parameters $R\left(S_{i},\;S_{j}\right)$ [$R^{\prime }\left(S_{i},\;S_{k}\right)$] are the equilibrium bond lengths between two nn nodes i and j (resp. i and k), which depend on the spin state and the nature (SCO or defect) of the connected sites. Owning to the square symmetry of the lattice (see Figure 1), we simply take, $R^{\prime }\left(S_{i},\;S_{k}\right)=\sqrt{2}R\left(S_{i},\;S_{k}\right)$. The different spin and lattice configurations of nn and nnn are summarized in Table 1. There, $R_{HH}$($R_{HH}^{\prime }$), $R_{LL}$($R_{LL}^{\prime }$) and $R_{HL}$($R_{HL}^{\prime }$) correspond to the nn (nnn) equilibrium bond lengths between HS-HS, LS-LS and HS-LS sites, respectively. Similarly, $R_{0H}$($R_{0H}^{\prime }$), $R_{0L}$($R_{0L}^{\prime }$) and $R_{00}$($R_{00}^{\prime }$) represent the respective nn (nnn) equilibrium bond length distances of HS-defect, LS-defect and defect-defect distances, respectively.

### 2.1. The General Expression of the Equilibrium Lattice Distance and Model Parameter Values

After a careful analysis of the constraints of Table 1 on the expressions of the bond lengths for the different spin configurations, we have been able to find a general formula Equation (2) linking the equilibrium bond length, $R\left(S_{i},\;S_{j}\right),$ with the spin states $\left(S_{i},\;S_{j}\right)$. This kind of expression is very useful and allows a better and more efficient Monte Carlo (MC) coding for the numerical simulations. The latter is written as:

(2) $$R\left(S_{i},\;S_{j}\right)=R_{HL}+\frac{\delta_{R}}{4}\left(S_{i}+S_{j}\right)S_{i}S_{j}+R_{00}\left(S_{i}^{2}-1\right)\left(S_{j}^{2}-1\right)\;+\frac{1}{2}{\left(S_{i}^{2}-S_{j}^{2}\right)}^{2}\left[\left(S_{i}+S_{j}+1\right)R_{0H}-\left(S_{i}+S_{j}-1\right)R_{0L}\right],$$

where $\delta_{R}=R_{HH}-R_{LL}$ is the lattice misfit between the pure (non-diluted) LS and HS phases. The reader can easily check that all configurations of Table 1 are included in this formula.

Throughout this report, we will consider the following relations between the equilibrium distances, $R_{HL}=\frac{R_{HH}+R_{LL}}{2},\;R_{0H}=\frac{R_{00}+R_{HH}}{2}$ and $R_{0L}=\frac{R_{00}+R_{LL}}{2}$, which decreases the number of free parameters from six to three (see Table 1). By fixing $R_{HH}$ and $R_{LL}$, only one free parameter, $R_{00}$, remains which will be used as a variable to monitor the thermal properties of the diluted SCO materials.

The following parameter values, derived from the experimental literature, are used in the present model. The ligand–field energy is taken constantly throughout the study as Δ=450 K [73] and is assumed as being independent of the presence of the defect, whose effect is then purely elastic. The latter value leads to an enthalpy change $\Delta H=3.5\;\mathrm{kJ}\cdot {\mathrm{mol}}^{-1}$, in good agreement with the experimental data available in the literature [74]. For the degeneracy ratio, we take the usual value, g=150 [75] leading to a molar entropy change at the transition $\;\Delta S=N_{A}k_{B}lng=41.63\;J\cdot K^{-1}\cdot {\mathrm{mol}}^{-1}$, $N_{A}$ being the Avogadro number. Similarly, we assume the degeneracy ratio as independent of the dilution effects as well as on temperature, as previously indicated. Within these values, the transition temperature is evaluated to, $T_{eq}=\frac{\Delta H}{\Delta S}=\frac{\Delta }{k_{B}lng}≃90\;K$ for the pure SCO lattice.

The values of the nn equilibrium distances in the LS and HS states are, respectively taken as equal to R(−1,−1)=1 nm and R(+1,+1)=1.2 nm, which leads for the HL configuration to $R\left(+1,-1\right)=\frac{R\left(+1,+1\right)+R\left(-1,-1\right)}{2}=1.1\;\mathrm{nm}.$ As already stated, the equilibrium nnn distances are simply considered as $\sqrt{2}R\left(+1,+1\right)=1.7\;\mathrm{nm},\sqrt{2}R\left(-1,-1\right)=1.4\;\mathrm{nm}$ and $\sqrt{2}R\left(+1,-1\right)=1.55\;\mathrm{nm}$ for HS-HS, LS-LS and HS-LS sites, respectively.

The nn molecules of the lattice interact elastically with the elastic constants $A=24,000\;{\mathrm{Knm}}^{-2}$, and we take for the nnn sites, B=0.3A, whose role is to maintain the stability of the 2D lattice. The above values of A and B lead to a bulk modulus, $E≃\frac{\left(A+2B\right)}{R}=6$ GPa, which is in quite fair agreement with data of the SCO literature [76,77] ranging between 5 and 20 GPa.

### 2.2. Simulations Method

The Hamiltonian (1) is solved by MC simulations. First, we generate a 2D square lattice with the bond lengths of the HS phase. The initial lattice coordinates of a site at the position (i,j) are $X\left(i,j\right)=R_{HH}\left(i-1\right)$ and $Y\left(i,j\right)=R_{HH}\left(j-1\right)$.

One of the critical points, when we perform a metal dilution relating to the positions of the defects in the lattice, let us consider a lattice with N nodes and fixed concentration, p, of defects. Then, there will be $\frac{N!}{\left(Np\right)!\left(N-Np\right)!}$ possible lattice configurations for the spatial distribution of the Np defects among the N lattice sites. At the level of one single crystal, the defect positions emerging from the chemical synthesis of the material are definitely fixed (we neglect diffusion effects). Therefore, one can draw randomly the defect positions and proceed to the simulations with the selected configuration.

However, it is clear that the results will depend on the spatial distribution of the dopants in the lattice. Thus, the results of the spin transition phenomenon may differ from one diluted microcrystal to another, even taken from the same batch. In the case of magnetic studies, for example, a large number of SCO powder particles or microcrystals is used. The emerging magnetic response is then that of the average responses of several particles, having more or less the same concentration of dopant but with different spatial distribution from one particle to another. In this work, we did not consider the possible situation where the defects may prefer, for affinity reasons, to be aggregated. The interactions between the defects (except elastic ones) are then ignored.

Along the simulations, several spatial distributions of the defects are generated randomly for each fixed dopant concentration. Once the defect positions are decided, they are fixed throughout the simulations. To ensure the random character of the spatial distribution of defects, using different random seeds we generate several spatial defect distributions at fixed rates of dilution. Then, we study the thermodynamic properties of each lattice configuration. At the end, the results are averaged on the ensemble of the studied lattices.

In practice, the stochastic algorithm of MC simulations is performed as follows. For a site “i” randomly selected with spin ($S_{i}=0,\pm 1)$ and position$\;{\vec{r}}_{i}$, a new spin value$\;S_{i}^{\prime }(=-S_{i}$) is set without a position change. This new spin value is accepted or rejected by the usual Metropolis criterion. It is worth noticing that we do apply the usual Metropolis criterion, even for the defect for which the spin flip does not change its total energy. Whatever the result of the spin flip attempt (the new spin value is accepted or rejected), the lattice positions are relaxed mechanically by a slight motion of each node (randomly selected) with a quantity $||\delta {\vec{r}}_{i}||\ll ||{\vec{r}}_{ij}||$, where $||{\vec{r}}_{ij}||$ is the distance between nn. This procedure is repeated 100 times so as to reach the mechanical equilibrium. Here, the reader can realize the importance of Equation (2), which allows the calculation of the equilibrium distance for any spin value (0,+1 or −1). In the present study we take the amplitude of the random displacements as δr=0.02 nm. This value remains quite small compared to the LS equilibrium distance $R_{LL}=1$ nm. Next, we randomly select a new site, and we repeat the procedure for all lattice sites. Once all the nodes of the lattice are inspected for the spin change, we define such a step as the unit of Monte Carlo step “MCS”. In these simulations, 1000 MCS are used to reach the thermal equilibrium without storing the data. For each MCS, the lattice is visited 10×N times to ensure the mechanical equilibrium, which means that each node is moved $10^{4}\times N$ times. The same number of MCS is used to average the physical quantities, like the high spin fraction,$\;n_{HS}$, defined as $n_{HS}=\frac{1+\langle S_{i}\rangle }{2}$, the average lattice bond length$\;\langle r\rangle =\frac{\sum_{ij}\left|r_{ij}\right|}{N_{nn}}$, where $\langle S_{i}\rangle =\frac{\sum_{i}S_{i}}{N}$ and $N_{nn}\approx 2N$ is the number of nn sites.

## 3. Results and Discussion

We have considered a square lattice (N=40×40) containing active spin crossover atoms and randomly distributed inactive defects with a fixed concentration, p. In addition to the concentration of defects, which is a natural relevant control parameter, we use the equilibrium bond length, $R_{00}$, between nn defects, chosen in the interval $\left[R_{LL},\;R_{HH}\right]$, to tune the thermal properties of this diluted SCO lattice.

### 3.1. Thermal and Structural Properties for $R_{00}=R_{LL}$

**Figure 2:**
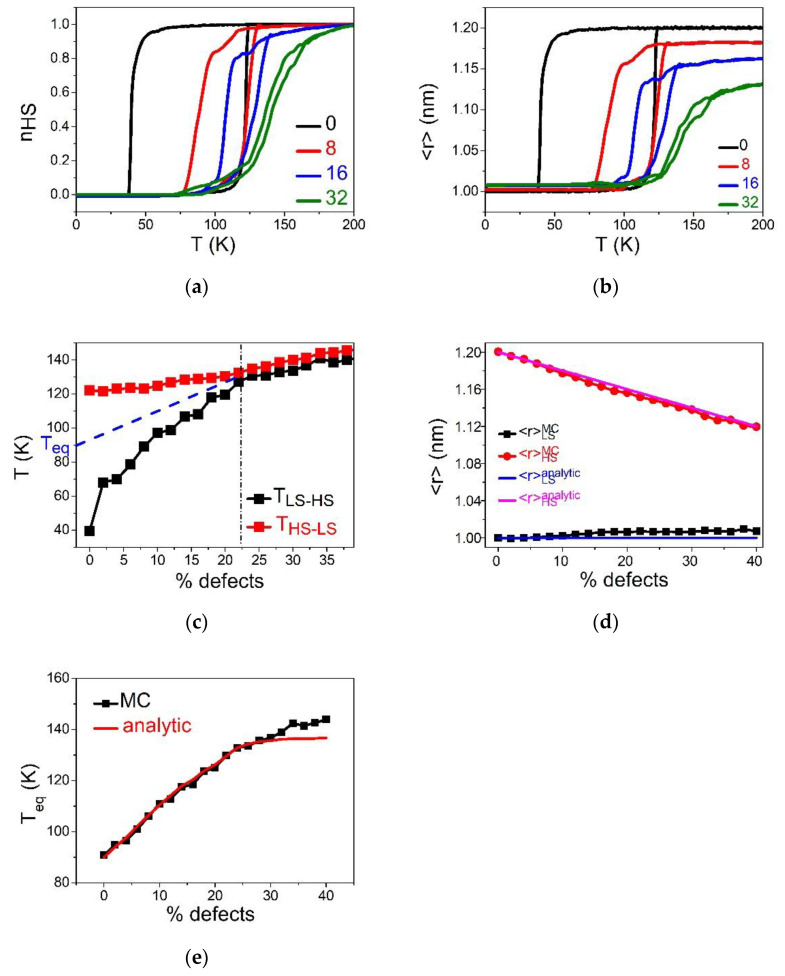
Thermal dependence of (**a**) the HS fraction, $n_{HS}$ and (**b**) average bond lengths 〈r〉, for different concentrations of defects when$\;R_{00}=R_{LL}$. (**c**) Evolution of transition temperatures $T_{LS\to HS}$ and $T_{HS\to LS}\;$from LS to HS (resp. HS to LS) as a function of percentage of defects. (**d**) Variation of lattice parameter $<r{>}_{LS}$ and $<r{>}_{HS}$ at low and high temperature, respectively as a function of defects concentration. (**e**) Comparison between MC and analytic equilibrium temperature, $T_{eq}$. See text for more explanations.

This section is devoted to the analysis of the effect of the concentration of dopant on the thermal and structural properties of an SCO lattice. Concentrations of defects going from 1% to 30% are considered with a defect–defect nn equilibrium distance equal to that of LS. This situation corresponds to the substitution of Fe by an inactive metal with an ionic radius equal to that of Fe in the LS state. As a consequence, it is expected that the defect favors structurally the LS lattice. To avoid elastic frustration problems, we started all simulations from the low-temperature region by preparing the lattice with the bond lengths of the LS state. Therefore, in the LS state, the lattice has zero elastic energy excess since all nn distances are equal to the equilibrium bond length, $R_{LL}$. The temperature is then varied from 1 to 200 K on heating and cooling, with a temperature step of  1 K. As already explained, the simulation is conducted with ten generated random lattices having the same concentration of dopant. Then, the physical quantities of interest are averaged over this sample. The obtained thermal dependencies are presented in Figure 2.

Figure 2a,b, summarizing the respective temperature evolution of the HS fraction, $n_{HS}$ and average bond length, 〈r〉, show that the pure SCO lattice (0% defects) exhibits an abrupt spin transition with hysteresis width (ΔT=80 K). When the concentration of defects increases, the cooperativity of the SCO reduces, as clearly seen in the reduction in the thermal hysteresis width. At the threshold value of defect concentration, $p_{c}=0.225$, and the first-order spin transition changes into a gradual transition, which corresponds to a simple Boltzmann population between two-degenerate levels. Figure 2c, depicting the phase diagram, p-T, shows that beyond the critical value, $p_{c}$, the two branches of the thermal hysteresis join and overlap, while keeping a linear increase with the concentration of defects. On the other hand, the equilibrium transition temperature, defined as $T_{eq}=\frac{T_{HS\to LS}+T_{LS\to HS}}{2}$, shifts towards high-temperature regions as the concentration of defects increases. This fact confirms that the small size dopant induces a positive pressure in the lattice, something which delays the LS to HS spin transition on heating and precipitates the HS to LS conversion on cooling. This behavior is in good agreement with the data of Figure 2c, showing a linear behavior of the upper, $T_{LS\to HS}$, and lower, $T_{HS\to LS},$ switching temperatures, recalling the behaviors of thermal hysteresis of SCO materials under-external pressure [5]. The remarkable difference in the slopes of $T_{LS\to HS}$ and $T_{HS\to LS}$, as function of the concentration of defect, is attributed to the kinetic effects of the MC procedure. Indeed, the switching temperature corresponds to the occurrence of a spinodal point, that is the point at which the lifetime of the metastable state falls down, which is crucially temperature-dependent. Consequently, the lower transition temperature $T_{HS\to LS},$ will be more sensitive to internal pressure than $T_{LS\to HS},$ which itself is located in a region where thermal fluctuations are already dominant.

To better understand these behaviors, we monitored the thermal variation of the average bond length, 〈r〉, and the dependence for different $R_{00}$ values. The results are given in Figure 2b. First of all, it is interesting to remark on the clear difference in the shape of hysteresis loops of $n_{HS}\left(T\right)$ and 〈r〉(T). In particular, Figure 2b reveals the presence of a monotonous decrease in the average HS bond length at high-temperature conditions (200 K for example), while this effect is absent in $n_{HS}\left(T\right)$ (Figure 2a). Thus, although $<r{>}_{HH}$ becomes lower than $R_{HH}$ when increasing p values, the spin system still reaches the full HS state (see Figure 2a). On the other hand, the absence of an effect in the LS state is easily understandable, since $R_{00}$ is chosen here as being equal to $R_{LL}$.

One of the important consequences of the reduction in the average HS bond length is the decrease in the lattice misfit between the HS and the LS phases. Knowing that the strength of the elastic interactions depends on the quantity, $\left(A+2B\right){\left(<r{>}_{HS}-<r{>}_{LS}\right)}^{2}$, one can easily understand that by decreasing $<r{>}_{HS}$. This in turn becomes smaller than $R_{HH}$, the steepest character of the thermal hysteresis for relatively small concentrations of defects transforms to bent hysteresis, with lower slopes of the heating and cooling branches.

#### 3.1.1. Analytical Study through a Homogenous Elastic Lattice

Our goal here is to understand the effect of the dopant’s concentration on the behavior of the average lattice bond length in the low- and high-temperature limits. For that, we perform an analytical study, based on the minimization of the expression of the total energy (elastic + electronic) in the HS and LS states with respect to the lattice parameter for a fixed concentration of defect. The calculation is conducted assuming a homogeneous lattice with a unique bond length, x. The energy densities of the HS and LS states, derived from Hamiltonian, (2) write,

(3) $$\begin{array}{ccc} \frac{E_{HS}^{tot}}{N} & = & \left(\Delta -k_{B}Tlng\right)\left(1-p\right)\\ & + & \left(A+4B\right)[{\left(1-p\right)}^{2}{\left(x-R_{HH}\right)}^{2}+p\left(1-p\right){\left(x-R_{0H}\right)}^{2}\\ & + & p^{2}{\left(x-R_{00}\right)}^{2}], \end{array}$$

(4) $$\begin{array}{ccc} \frac{E_{LS}^{tot}}{N} & = & -\left(\Delta -k_{B}Tlng\right)\left(1-p\right)\\ & + & \left(A+4B\right)[{\left(1-p\right)}^{2}{\left(x-R_{LL}\right)}^{2}+p\left(1-p\right){\left(x-R_{0L}\right)}^{2}\\ & + & p^{2}{\left(x-R_{00}\right)}^{2}]. \end{array}$$

The mechanical equilibrium is obtained by minimizing the total energy with respect to the x variable in the HS and LS phases, which leads to the following general expressions of relaxed bond lengths:

(5) $$x_{relax}^{HS}=\frac{p²R_{00}+p\left(1-p\right)R_{0H}+\left(1-p\right)²R_{HH}}{p+\left(1-p\right)²}\;,$$

(6) $$x_{relax}^{LS}=\frac{p²R_{00}+p\left(1-p\right)R_{0L}+\left(1-p\right)²R_{LL}}{p+\left(1-p\right)²}.$$

Using now $R_{00}=R_{LL}$ and $R_{0H}=R_{HL}$, we obtain:

(7) $$x_{relax}^{LS}=R_{LL}\;and\;x_{relax}^{HS}=R_{HH}-\frac{\delta_{R}}{2}\frac{p\left(p+1\right)}{p+{\left(1-p\right)}^{2}}$$

Figure 2d summarizes the comparison between the analytical and MC data for the dopant concentration-dependence of the bond lengths, showing a good agreement between the two set of results.

To determine the analytical expression of the transition temperature, we first define the value of the relaxed bond length at the transition temperature, $T_{eq},$ as $x_{1/2}=\frac{x_{HS}^{relax}+x_{LS}^{relax}}{2}$. Then, we calculate $T_{eq}$ using the relation $E_{tot}^{HS}\left(x_{1/2}\right)=E_{tot}^{LS}\left(x_{1/2}\right)$, which remains valid as long as we neglect the configurational entropy of spins contribution. This leads to:

(8) $$x_{1/2}=R_{HL}-\frac{p\left(1+p\right)}{\left[p+{\left(1-p\right)}^{2}\right]}\delta R,$$

(9) $$T_{eq}\left(p\right)=T_{eq}\left(0\right)+\left(A+4B\right)\frac{2p\delta R}{k_{B}lng}\left[\frac{\left(1+p\right)\left(2-p\right)}{\left[p+{\left(1-p\right)}^{2}\right]}\delta R+R_{LL}\right].$$

Figure 2d,e present the p-dependence of $x_{1/2}\;$ (that is 〈r〉) and $T_{eq}$ obtained with Equations (8) and (9) and MC simulations. It appears that, while a good agreement is obtained for the evolution of 〈r〉 (Figure 2d), that of the temperature (Figure 2e) is subtler. Indeed, a fair agreement exists between the analytical and MC data for p values ranging between 0 and 30%, meeting the assumption of the homogenous concentration of defects. However, a discrepancy emerges for strong concentrations of defects. This is attributed to the fact that the assumption of homogenous distribution of dopants, used in the analytical approach, is less valid for p>0.3. Overall, the increase in the transition temperature with dopant concentration is clearly due to $R_{00}=R_{LL}$, which clearly plays the role of an internal positive pressure in the lattice.

#### 3.1.2. Spatiotemporal Aspects of the Phase Transition

**Figure 3:**
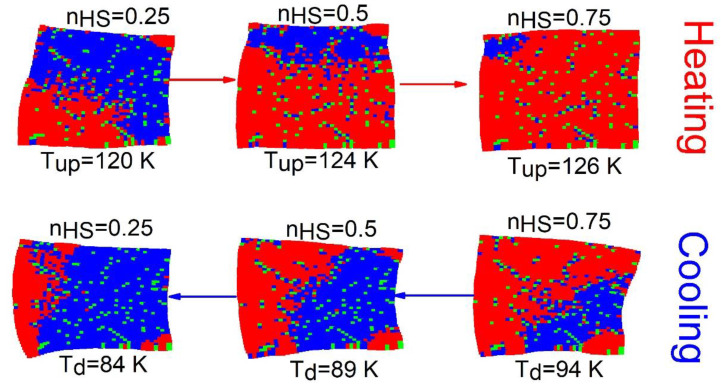
Selected crystal snapshots showing both electronic and macroscopic distortion configurations during the heating and cooling processes starting from LS state for a concentration,  p=8% of defects and $R_{00}=R_{LL}$. The red (blue) dots represent the HS (LS) sites and green dots stand for the dopant sites.

The spatiotemporal studies of the thermoinduced spin transition concern the analysis of the mechanisms of formation (nucleation and growth) of HS and LS domains, along with the cooling and heating branches of the thermal hysteresis. For that, the spatial configurations of the spin-lattice states are stored at selected temperatures. Figure 3 displays a set of snapshots of the spin and lattice configurations along the thermal hysteresis loop of Figure 2a. Here, red, blue and green dots represent the HS, LS and dopant sites, respectively.

Figure 3 reveals, that upon heating ($n_{HS}=0.25$) for a crystal with 8% of defects, the HS phase grows exclusively and quite easily from the free corners (due to surface effects), and then extends over the whole crystal, by avoiding the defects. It is also remarked that the spatiotemporal aspects of the HS→LS transition (on cooling) are quite different from those observed on heating. Indeed, on cooling the LS phase mainly nucleates around the defects and deploys over the entire crystal. In contrast, on heating the HS species grows far from the defects; these maintain their surrounding sites in the LS state, switching later at the end of the process.

One can also notice a significant lattice distortion near to LS (resp. HS) domains during the heating (resp. cooling) process. On the other hand, as the number of defects increases (p value), the proportion of active SCO centers reduces, and then the lattice distortion is expected to be reduced fairly, due to the reduction in the global volume change.

### 3.2. Thermal and Mechanical Properties for $R_{00}=R_{HL}$

**Figure 4:**
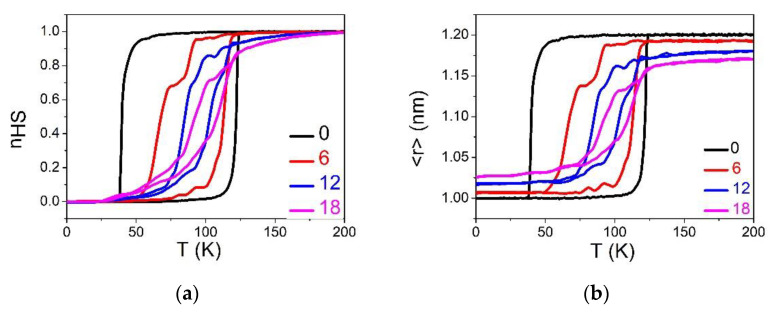

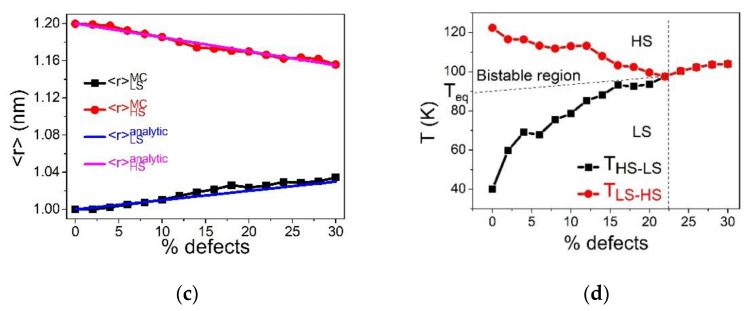
(**a**,**b**) Thermal dependence of the HS fraction, $n_{HS}\;$ and average bond lengths 〈r〉 for different concentration of defects when$\;R_{00}=R_{HL}$. (**c**) Variation of lattice parameter $<r{>}_{LS}$ and $<r{>}_{HS}$ at low and high temperature, respectively, as a function of defect concentration. Filled circles and squares are MC data, and full pink and blue lines are analytical predictions deduced from Equation (10). (**d**) Evolution of transition temperatures, $T_{LS\to HS}$ (resp. $T_{HS\to LS}$), from LS to HS (resp. HS to LS) as a function of the concentration of defects showing a weak p-dependence.

In this part, we study the case, $R_{00}=R_{HL}=\frac{R_{HH}+R_{LL}}{2},$ where the dopant is expected to have a “neutral” elastic role. The concentration of defects varied in the range of 1–30%. Since $R_{00}$ is neither equal to $R_{HH}$, nor to $R_{LL}$, the initial elastic lattice (HS or LS) will be frustrated. Consequently, it is important to make sure, during the simulation, that the lattice is well relaxed at each temperature step. So, starting from the relaxed structure of the crystal, we vary the temperature in the range 1–200 K on heating and cooling, with a thermal step of 1 K. The thermoinduced spin transition for the different concentrations of defect is illustrated in Figure 4 in terms of HS fraction, $n_{HS}$, and average lattice bond lengths, 〈r〉.

Similarly to panels (a) and (b) of Figure 2, Figure 4a,b show that the presence of defects reduces the cooperativity inside the SCO lattice, which changes from an abrupt thermal transition to gradual transition to a high concentration (≥24%). Here, the equilibrium temperature,$\;T_{eq}$, is slightly shifted towards higher temperature. To understand this decrease in cooperativity, we monitored the thermal variation of the average lattice parameters in Figure 4b. One can notice a difference in the shape of hysteresis loops of $n_{HS}$ and 〈r〉 at low and high temperatures. Indeed, increasing the proportion of defects results in an increase in $<r{>}_{LS}$ and as decrease in $<r{>}_{HS}$, as shown in Figure 4c. As a result, the global change in lattice parameter ($\delta \langle r\rangle =<r{>}_{HS}-<r{>}_{LS})$ between the HS and LS phases decreases linearly with p.

As already explained, the reduction in the lattice misfit between the LS and HS states causes a reduction in the strength of the elastic interactions. This effect is responsible for the long-range nature of the cooperative interactions in SCO solids. Thus, the decrease in δ〈r〉 as function of p results in the vanishing of the thermal hysteresis width on increasing the dilution rate. In other words, the defects prevent the propagation of the elastic strain and isolate the active SCO species, leading to a cut in the interaction paths.

The analytical dependence of $<r{>}_{HS}$ and $<r{>}_{LS}$ can be obtained by minimizing the total energy in the HS and LS states. Using expressions (5) and (6), we find for the present case:

(10) $$x_{relax}^{LS}=R_{HL}-\frac{\left(1-p\right)\left(2-p\right)}{p+{\left(1-p\right)}^{2}}\frac{\delta R}{4}\;\mathrm{and}\;x_{relax}^{HS}=R_{HL}+\frac{\left(1-p\right)\left(2-p\right)}{p+{\left(1-p\right)}^{2}}\frac{\delta R}{4}$$

For the relatively small p values, Equation (10) gives for relaxed LS and HS bondlengths, the respective expressions $x_{relax}^{LS}=R_{LL}+p\delta R/4$ and $x_{relax}^{HS}=R_{HH}-p\delta R/4$, which well fit with the MC data of Figure 4c.

On the other hand, from the equality, $E_{tot}^{HS}\left(x_{1/2}\right)=E_{tot}^{LS}\left(x_{1/2}\right)$, we easily obtain the expressions of the transition temperature, $T_{eq}$, and the corresponding value of the average lattice bond length, $x_{1/2}$:

(11) $$\;T_{eq}=\frac{\Delta }{k_{B}lng}\;\mathrm{and}\;x_{1/2}=R_{HL}.$$

Equation (11) indicates that we expect a constant equilibrium temperature, independent of the concentration of dopants. This is in quite good agreement with the MC results of HS fraction and 〈r〉 (Figure 4a,b), which show that the hysteresis loops are almost all centered around T=90 K for the different p values. In addition, Figure 4d, which summarizes the behavior the p-dependence of the limiting switching temperatures of the hysteresis, has an almost symmetrical shape. It is thus deduced that the MC equilibrium temperature, $T_{eq}$, slightly changes with p, in agreement with Equation (11). This trend is attributed to the neutral elastic character of the defect, whose size $R_{00}=R_{HL}$, is in the middle between those of HS and LS bond lengths.

#### Spatiotemporal Aspects

**Figure 5:**
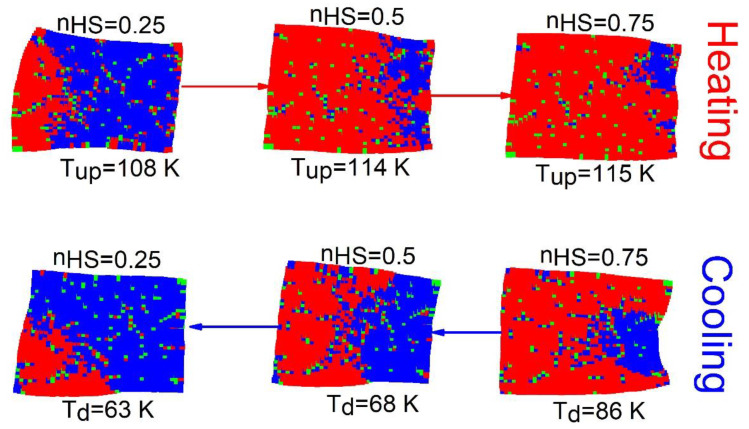
Snapshots of the crystal during the heating and the cooling processes starting from LS state for 6 % of defects corresponding to Figure 4a. The red (blue) dots are associated with HS (LS) sites, while the green dots represent the dopant. The nn defect–defect distance is $R_{00}=R_{HL}$.

Here, we investigate the spatiotemporal properties of the thermal hysteresis of Figure 4. The results are presented in Figure 5. We notice that upon heating (for $n_{HS}=0.25$) for a crystal with 6% of defects, the HS phase grows from the right corners and edges where the elastic energy is weaker. Then, the domains extend towards the center of the lattice with a clear interface, avoiding the regions with rich concentrations of defects, which convert only at the end of the process. Contrary to the previous case ($R_{00}=R_{LL}$), here the spatiotemporal feature of the HS→LS transition (on cooling branch) is quite similar to that of the heating (LS→HS) branch. Thus, this behavior confirms the neutral role of the defect, which does not favor the nucleation of one state over the other.

### 3.3. Thermal and Mechanical Properties for $R_{00}=R_{HH}$ and Emergence of HS Residual Fraction

Now, we analyze the special case where the equilibrium nn dopant–dopant distance is equal to nn bond length of the HS phase ($R_{00}=R_{HH}$), for various defect concentrations, p, going from 1 to 30%. Contrary to the case $R_{00}=R_{LL}$, here we start the simulations from the high temperature (i.e., HS) phase where the system is elastically relaxed. The system is then cooled from 200 to 1 K and then heated with a thermal step of 1 K.

**Figure 6:**
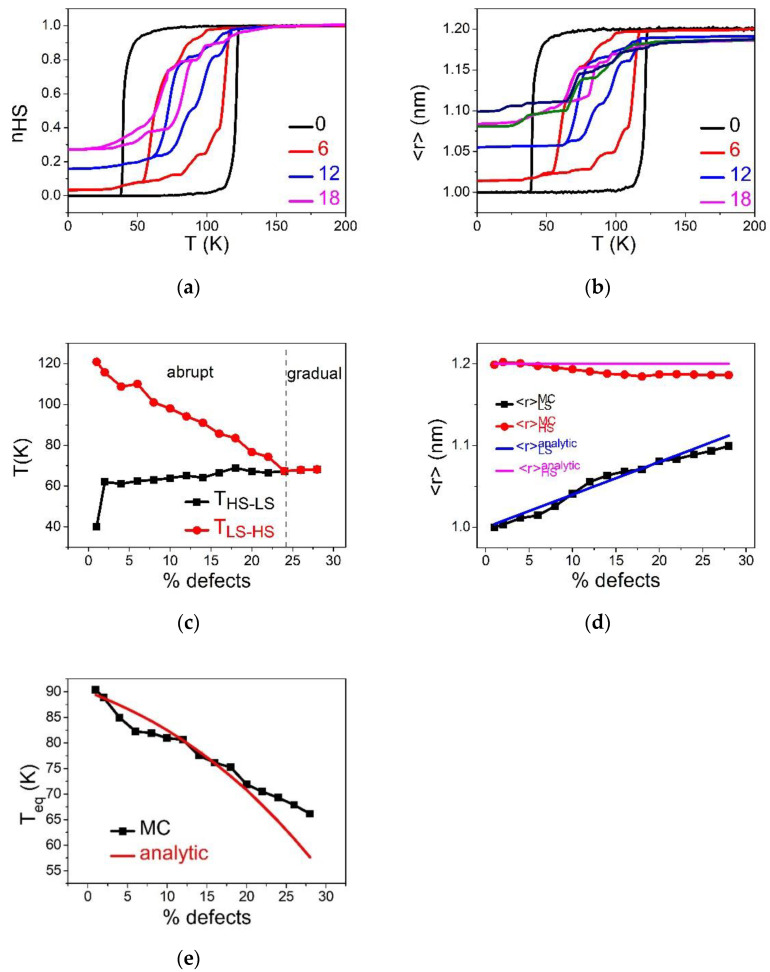
(**a**,**b**) Thermal dependence of the HS fraction, $n_{HS}\;$ and average bond lengths 〈r〉  for different concentration of defects when$\;R_{00}=R_{HH}$. (**c**) Evolution of transition temperatures $T_{LS\to HS}$ (resp. $T_{HS\to LS}\;$) from LS to HS (resp. HS to LS) as a function of defects concentration. (**d**) Variation of lattice parameter $<r{>}_{LS}$ (resp. $<r{>}_{HS})\;$at a low (resp. high) temperature, versus the concentration of defects. (**e**) Comparison between numerical and analytic equilibrium temperature, $T_{eq}$, as a function of p, showing a fair qualitative tendency.

The thermoinduced spin transitions for the different concentrations of defects are illustrated in Figure 6 in terms of $n_{HS}$ and average lattice bond lengths, 〈r〉, repectively.

One can notice from Figure 6a that, once again, the presence of defects reduces the cooperativity of SCO units leading to a gradual transition for defect concentrations bigger than 24%. This reduction in the thermal hysteresis width is now accompanied with the appearance of a residual HS fraction at a low temperature. The presence of this residual HS fraction, leading to incomplete spin transition at low temperatures and its increase with dopant concentration, is in direct line with the thermal variation of the lattice parameters. Indeed, Figure 6b presenting the thermal dependence of 〈r〉 reveals a significant increase in the average bond length distance at low temperature as p increases. The simple explanation is that, within the presently used equilibrium bond length value ($R_{00}=R_{HH}$) of the dopant lattice, the SCO lattice expands locally around the defect positions, particularly in the LS state. This creates a tensile stress which acts on the effective ligand field and favors the local nucleation of HS species around defect regions even at low temperature, when the local bond distances exceed the $R_{HL}$ value. A careful examination of the p-dependence of the upper and lower switching temperatures, presented in Figure 6c, demonstrates that almost only the HS→LS transition is affected by the dilution.

To compare the obtained numerical values of 〈r〉 at low and high temperatures with the analytical ones, we minimize the total energy in the HS and LS states, using Equations (5) and (6), for $R_{00}=R_{HH}$. The calculations lead to the following expressions of relaxed bond lengths,

(12) $$x_{relax}^{LS}=R_{LL}+\frac{p\delta_{R}}{2}.\frac{1+p}{p+{\left(1-p\right)}^{2}}\;and\;x_{relax}^{HS}=R_{HH}.$$

The expressions of the transition temperature, $T_{eq}$, and corresponding average bond length, $x_{1/2},$ obtained from the relation $E_{tot}^{HS}\left(x_{1/2}\right)=E_{tot}^{LS}\left(x_{1/2}\right)$ (Equations (3) and (4)), are given below:

(13) $$x_{1/2}=R_{HL}+\frac{p\left(1+p\right)}{\left[p+{\left(1-p\right)}^{2}\right]}\delta R,$$

(14) $$T_{eq}\left(p\right)=T_{eq}^{\left(0\right)}-\frac{\left(A+4B\right)}{8k_{B}lng}p\delta R\left[\frac{\left(1+p\right)\left(2-p\right)}{\left[p+{\left(1-p\right)}^{2}\right]}\delta R+R_{HH}\;\right].$$

The comparison between the MC results and the analytical predictions show a good qualitative agreement for the general tendencies, as depicted in Figure 6d,e, although some discrepancy exists for the transition temperature (Figure 6e). The latter is mainly due to the limitations of the validity of the homogeneous distribution of dopants assumed in the analytical approach.

#### Spatiotemporal Aspects

**Figure 7:**
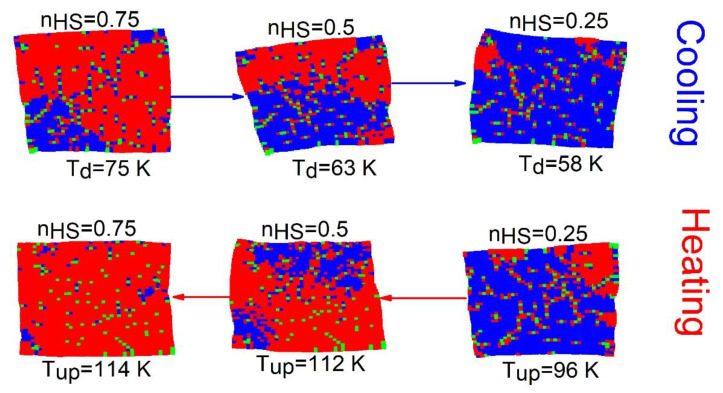
Snapshots of the crystal showing the nucleation and growth features of a diluted SCO lattice along the heating and cooling processes for p=6% of defects. The red (blue) dots are associated with HS (LS). The green sites represent the dopant sites. The nn defect–defect distance is, $R_{00}=R_{HH}$.

We have analyzed the presence of defect having the same size as that of HS ion on the spatiotemporal aspects of the thermoinduced spin transition. The obtained results are presented in Figure 7, where we display selected snapshots of the spin state configuration along the thermal hysteresis loop of Figure 6a.

One can notice from Figure 7 that, on heating from the LS state, the nucleation of the HS phase mainly starts around the defects thanks to the negative pressure they produce on the LS sites, since, $R_{0L}=\frac{R_{00}+R_{LL}}{2}=\frac{R_{HH}+R_{LL}}{2}=R_{HL}>R_{LL}$. This means that each LS site with a defect neighbor experiences a tensile stress, which helps with the emergence of HS species around the defects. Thus, the nucleation of the HS phase appears more or less as homogeneously appearing everywhere—in the center, the corners and the edges—due to a random spatial distribution of the defects. In contrast, on cooling, the LS phase tends to appear mostly from corners and edges, far from defects, and coalesce at the end of the process around the defect regions, which are the last to convert into the LS state. In all snapshots, we see that the lattice deforms along the transition, but these deformations remain less severe than those of the pure SCO solid.

### 3.4. Low-Temperature Relaxation of the Photo-induced Metastable HS State

The metastable HS state is produced at low temperature by a photo-excitation through an adequate wavelength by the so-called LIESST effect [67]. In addition, a rapid quenching (or flash cooling) from the high temperature thermodynamic stable HS phase may also lead to the generation of a quenched metastable HS phase at a low temperature. However, the microscopic nature of the latter is different from that of the photo-induced HS state [78,79,80]. Here, we aim to analyze the effect of dilution on the isothermal HS→LS relaxation process of a SCO system. For that, we investigate the temporal evolution of the HS fraction, $n_{HS},$ the average lattice parameter 〈r〉 as well as the spatiotemporal properties of the spin organization and lattice deformation along this isothermal relaxation process. Initially, the lattice of size, (N=40×40 ), is prepared in the HS state from the electronic ($S_{i}=+1)\;$and structural ($r_{ij}=R_{HH})\;$point of view at T=1 K. At this temperature, the prepared HS state is clearly metastable, and the LS state ($S_{i}=-1,\;r_{ij}=R_{LL})$ is the thermodynamic stable phase. Then, isothermal MC simulations are performed on both spin and position variables for various dopant concentrations, p, and defect sizes ($R_{00}$). The objective here is to enhance the understanding of the combined effects of p and $R_{00}$ on the features of the electro-elastic relaxation mechanism.

#### 3.4.1. Case of $R_{00}=R_{LL}$

**Figure 8:**
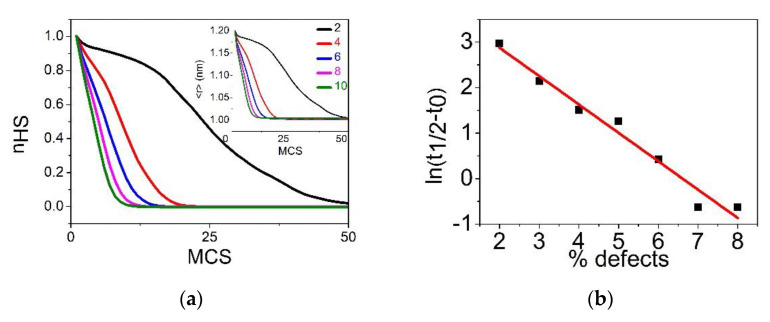

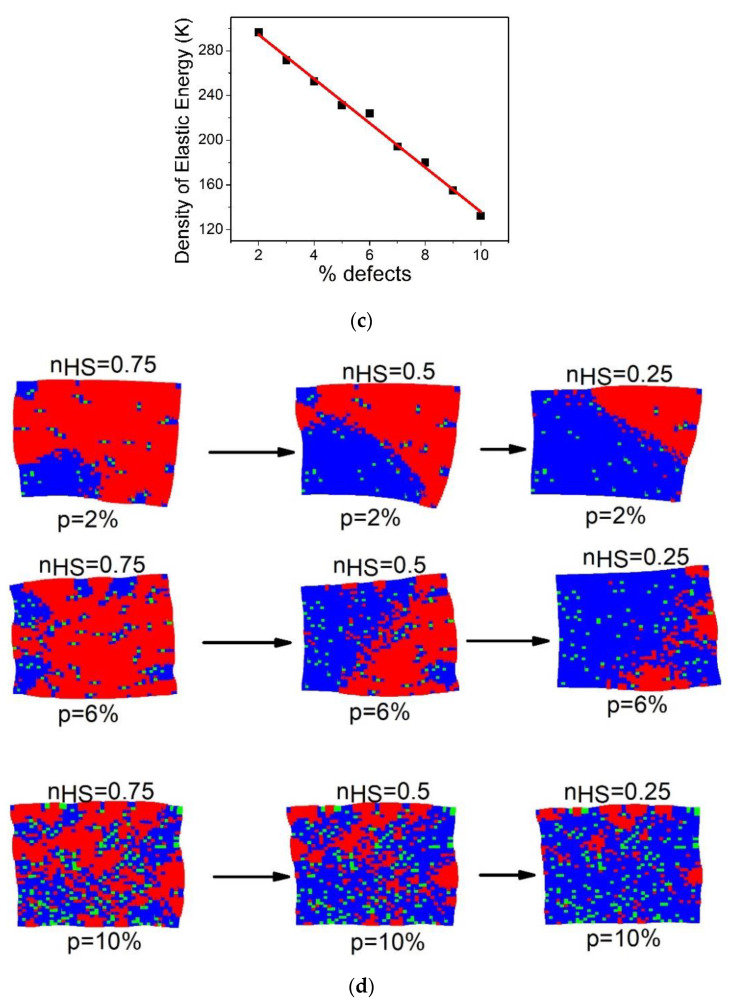
(**a**) Time dependence of the metastable HS fraction, $n_{HS}$, and average bond lengths 〈r〉 (given in inset) throughout the HS→LS relaxation for a square lattice of size 40×40. The data have been obtained by MC simulations at T=1 K for different concentration of defects: p=2% (black), 4% (red), 6% (blue), 8% (pink) and 10% (green). (**b**) Linear behavior of the logarithm of the lifetime, $ln\left(t_{1/2}-t_{0}\right)$, of the HS state as a function of p. (**c**) Density of elastic energy as a function of the concentration of defects. (**d**) Spatiotemporal configurations of the spin and lattice transformations during the HS→LS relaxation. Red (blue) area represents HS (LS) phases, and the green dots stand for the defect. From top to bottom: p=2%, 6% and 10%. Left to right:$\;n_{HS}=0.75$, 0.5 and 0.25.

In this section, we report the time dependence of the average HS fraction, $n_{HS},$ and lattice parameter, 〈r〉, during the relaxation from the metastable HS fraction at T=1 K for several defect concentration values ranging from p=2% to 10%. The obtained results for the relaxation of the HS fraction (resp. 〈r〉) are summarized in Figure 8a (resp. Inset of Figure 8a). First, we see that the shape of the relaxation curves changes from being sigmoidal (non-linear) for low p values to almost being exponential for p=10%. This behavior is clear evidence of the decreasing cooperative character of the system with dilution. On the other hand, the lifetime, $t_{1/2}$, of the metastable HS state, defined as the transit time at $n_{HS}=1/2$, is expected to grow exponentially as $t_{1/2}∼t_{0}+\alpha e^{\Delta E/kT}$ with the energy barrier ΔE. It is important to notice that ΔE contains two contributions. The first one is the electronic gap, $\Delta E_{elec}=E_{elec}^{LS}-E_{elec}^{HS}=-2\left(\Delta -k_{B}Tlng\right)\left(1-p\right)$, which is negative, owing to the stable nature of the LS state at 1 K. The second one is elastic, $\Delta E_{elast}=E_{elast}^{LS}-E_{elast}^{HS}$, which is positive, and constitutes the energy barrier facing the switching between HS and LS states. These two competing contributions are well identified in Equations (3) and (4). Thus, $\Delta E_{elec}$ is the driving force of the relaxation of the HS sites towards the LS state, while $\Delta E_{elast}$ opposes to this spin switching. Overall, the profile of the relaxation curve is due to the competition between these two contributions. In a homogeneous system, with low dopant concentrations (1 to 10%), both energy gaps are expected to linearly depend on the dopant concentration, p, as shown in Equations (3) and (4). Interestingly, Figure 8b shows that the $ln\left(t_{1/2}-t_{0}\right)$ linearly decreases with p, which corroborates the assumption of the linear p-dependence of $\Delta E_{elast}$. The latter is demonstrated in Figure 8c, which reports the linear decrease in the elastic energy with the dopant concentration, while this is obvious for the electronic energy.

Figure 8d illustrates the electro-elastic configuration of the lattice during the relaxation process for three p values: 2%, 6% and 10%. In the case of p=10%, the LS phase appears dispersed over the lattice, appearing everywhere, even in the center, which explains the exponential character of the corresponding relaxation curve. This rapid relaxation towards the LS state is induced by the presence of a significant concentration of dopants with size $R_{00}=R_{LL}$, leading to shrink the lattice. This elastic effect acts as an external non-uniform pressure affecting all HS sites. In contrast, for p=6%, we observe that the nucleation of LS domains mainly starts from corners and edges, and then propagates toward the center. Upon decreasing p, the number of LS nucleation centers decreases until a single macroscopic domain nucleation mechanism emerges for  p=2%. The latter recalls the results obtained for non-diluted SCO systems, leading to sigmoidal relaxation curves of the HS fraction, characteristic of strong cooperative systems.

#### 3.4.2. Case of $R_{00}=R_{HL}$

**Figure 9:**
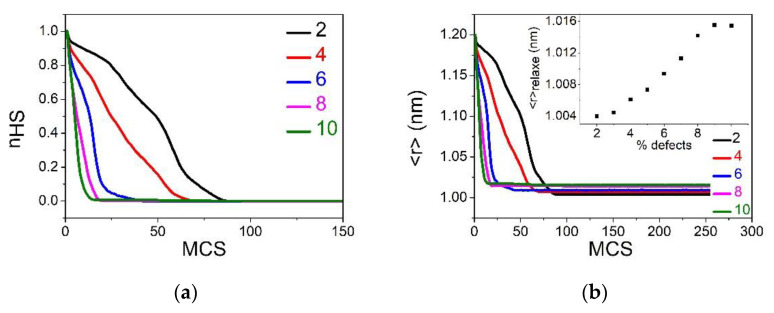

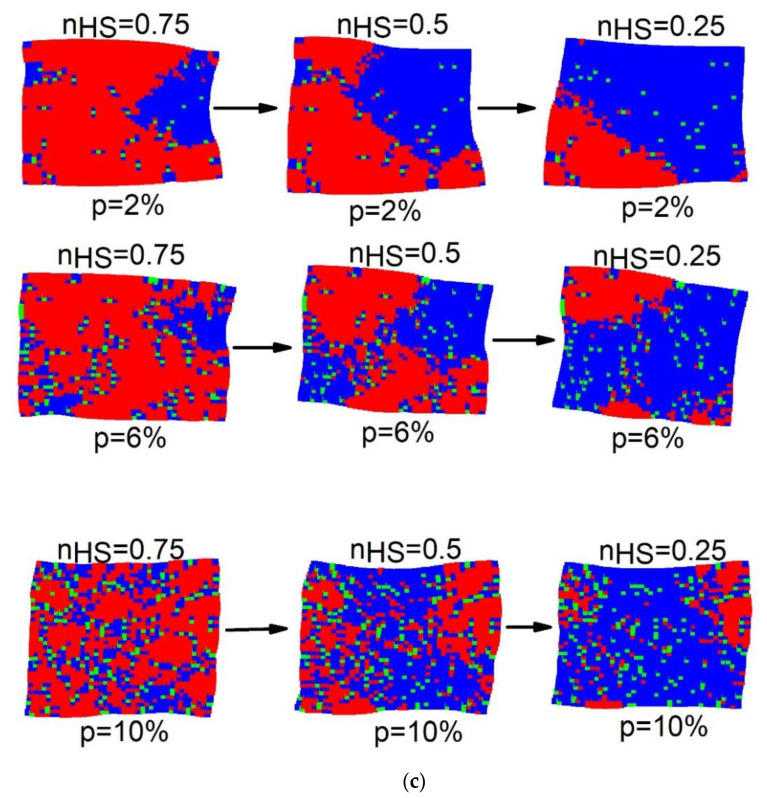
(**a**) Time dependence of the metastable HS fraction, $n_{HS}$, along the HS→LS relaxation for a lattice 40×40. The data obtained from MC simulations at T=1 K. The five curves correspond to the following concentrations of defects: p=2% (black), 4% (red), 6% (blue), 8% (pink) and 10% (green). (**b**) Corresponding temporal evolution of the average bond length, 〈r〉. Inset: relaxed average bond length in the LS phase as function of p, showing a quasi-linear behavior. (**c**) Spatiotemporal configurations of the spin and lattice transformations during the HS→LS relaxation. Red (blue) area represents HS (LS) phases and green dots stand for the defect. From top to bottom: p=2%, 6% and 10%, respectively. Left to right: $n_{HS}=0.75,$ 0.5 and 0.25, respectively.

The relaxation is now performed with defects, assumed to be elastically neutral, since their size, $R_{00}=R_{HL}=\frac{R_{HH}+R_{LL}}{2}$, is between those of HS and LS. The simulations are conducted for different concentrations of defects and we proceed by starting from the metastable HS state, characterized by $\langle r\rangle =R_{HH}$ and $S_{i}=1$ ∀ i, i.e., $n_{HS}=1$. The temporal dependence of the HS fraction, $n_{HS}$, and average bond length 〈r〉, are summarized in Figure 9a,b, respectively. As for the results of Figure 8, the lifetime of the metastable HS state, decreases with the concentration of dopants and the HS fraction reaches the value 0 (that of LS state) at the end of the relaxation. In addition, the shape of the relaxation curves changes from sigmoidal to exponential as p increases. On the other hand, the curves of the average bond length, 〈r〉, displayed in Figure 9b, show a slight difference, compared to those of $n_{HS}$. Indeed, at the end of the relaxation, the stationary value of $<r{>}_{LL}$ remains bigger than the average bond length, $R_{LL}$, of the LS state, although the corresponding value of $n_{HS}$ is zero.

The p-dependence of $<r{>}_{LL}$ in the LS state, presented in the inset of Figure 9b shows a quasi-linear dependence, which means that the average lattice bond length in the relaxed LS phase increases with the dilution rate. Consequently, although the spin system converts to the full LS state, a tensile strain does remain in the lattice in the LS phase due to the defect size. To understand this point, let us briefly discuss the analytical expression of $<r{>}_{LL}$. If we consider the SCO lattice as an effective homogeneous medium, then the average lattice parameter in the LS phase is around $<r{>}_{LL}≃\left(1-p\right)R_{LL}+p^{2}R_{00}+p\left(1-p\right)\frac{R_{00}+R_{LL}}{2}$. For, $R_{00}=R_{HL}$, and neglecting all contributions on $p^{2}$ (too small), one finds $<r{>}_{LL}≃R_{LL}+p\frac{\delta R\;}{4}$, which confirms the linear p-dependence of $<r{>}_{LL}$ found in the MC simulations.

Figure 9c collects some selected snapshots of the electro-elastic configuration of the lattice during the relaxation process for different p values. As for the case of Figure 8d, the relaxation is accompanied with a significant lattice distortion and single-domain nucleation process appears for low dilution rates (2%), while homogenous nucleation is dominant for the higher dilution rate (10%).

#### 3.4.3. Case of $R_{00}=R_{HH}$

**Figure 10:**
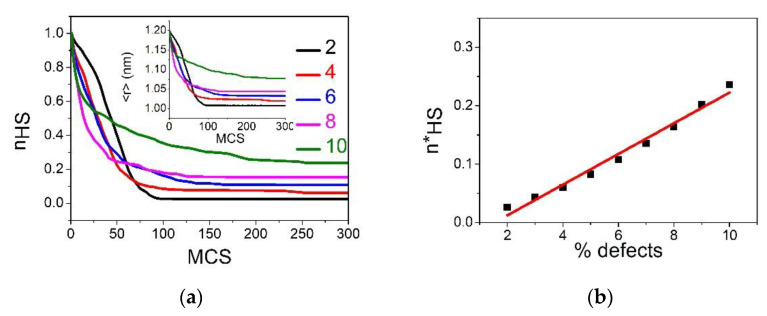

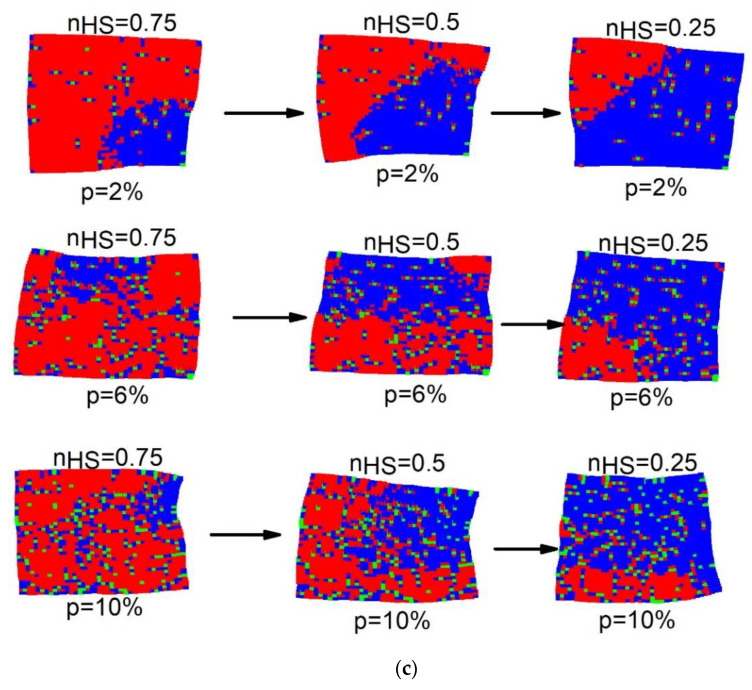
(**a**) Time dependence of the metastable HS fraction, $n_{HS}$, along the HS→LS relaxation. The data are obtained by MC simulations at T=1 K, on a square lattice of size 40×40, for the following concentrations of defects: p=2% (black), 4% (red), 6% (blue), 8% (pink) and 10% (green). Inset: corresponding time dependence of the average bond lengths 〈r〉. (**b**) Residual HS fraction in the relaxed regime, $n_{HS}^{*}$, derived from panel (a), as a function of concentration of defects. (**c**) Spatiotemporal configurations of the spin and lattice during the HS→LS relaxation. Red (blue) squares represent HS (LS) phases while green square stand for the dopant. From top to bottom: p=2%,6% and 10%, respectively. Left to right: $n_{HS}=0.75$, 0.5 and 0.25, respectively.

Now we examine the situation where the size of the dopant is equal to that of the HS ion. In this case, the photo-induced metastable HS state (all SCO spins are set to +1) is free from an elastic strain, since all nn distances are equal to $R_{HH}$. Consequently, the initial diluted HS state has an elastic energy equal to zero. It is then expected that the dopant will have two roles: first it stabilizes the HS phase, and at some threshold concentration it will destroy the elastic interactions between the SCO atoms, as for the previous cases. The time dependence of the average HS fraction, $n_{HS},$ during the relaxation from the metastable HS is given in Figure 10a and that of 〈r〉 is presented in the inset of Figure 10a. First, we remark that the shapes of the relaxation curves are very different from those of the two previous studied cases ($R_{00}=R_{LL}$ and $R_{00}=R_{HL}$). Indeed, except the case p=2%, for all other concentrations, the HS fraction $n_{HS}$ does not reach the value 0 at a long time, which means that a residual HS fraction does subsist in the lattice in the stationary state. Interestingly, this residual fraction, denoted as $n_{HS}^{*}$, linearly increases with the concentration of dopant, as depicted in Figure 10b. This behavior is also observed on the structural order parameter, 〈r〉 (see inset of Figure 10a), which does not reach the equilibrium bond length of the LS state, $R_{LL}$, even for weak p values. Consequently, the lifetime of the metastable HS state increases with  p. This is well explained through the elastic role of the dopant, which expands the lattice, thus preventing its efficient shrinking along the HS → LS relaxation. This negative pressure maintains a global tensile stress that allows the HS state to survive in the immediate vicinity of the dopants. As a result, the SCO atoms close to the dopant stay in the HS state later, compared to those surrounded by SCO sites. Thus, the ligand fields of the SCO sites are renormalized by their nn or nnn dopants, and this renormalization fluctuates from one site to another due to the fluctuations of the local concentration of dopant. At the end, the shape of the relaxation curve of the HS fraction switches from sigmoidal for low p values to stretched exponential, as p increases. The latter is the signature of the decrease in the strength of the interactions combined with disorder arising from the random spatial distribution of the dopants inside the SCO lattice.

Figure 10c illustrates the electro-elastic configuration of the lattice during the relaxation process for different dopant concentration values. Contrary to the previous cases, now the LS phase emerges as a single macroscopic domain mechanism for all concentrations, even for those leading to stretched exponential relaxation shapes (see Figure 10a). In this case, the switching to LS of the HS sites close to defects are delayed to longer relaxation times, due to the negative pressure induced by the dopant, which decreases the effective ligand field of the SCO sites.

## 4. Conclusions

We investigated by means of MC simulations and an analytical approach the thermal and the non-equilibrium properties of diluted SCO materials, using an electro-elastic model combining spins and deformations variables. The present study focuses on the effects of metal dilution on the bistability of SCO materials. Among them, we examine the impact of dilution on the thermal hysteresis as well as on the macroscopic nucleation and growth mechanisms taking place along the thermoinduced phase transition. For that, we analyze the dependence of the transition temperature and the thermal hysteresis width along with the concentration of dopant. To prove the important elastic role of the dopant, we consider three possible sizes for the dopant–dopant distance ($R_{00}$) in the lattice: $R_{00}(=R_{LL}$, $R_{HL}$, $R_{HH}$), corresponding, respectively to those of LS-LS, HS-LS or HS-HS sites. We find that, when $R_{00}=R_{LL}$, the increase in the concentration of defects changes the thermoinduced spin transition of the crystal from abrupt to gradual, with a shift of the transition temperature towards lower temperatures [55,61]. It is then found that the dilution affects both the cooperativity of the system since the hysteresis vanishes and the effective ligand field, due to the lowering of the transition temperature. When, $R_{00}=R_{HL}$, the thermal hysteresis vanishes on increasing the concentration of defects above the threshold value, p=24%, while the transition temperature remains invariant. In the last case, corresponding to $R_{00}=R_{HH}$, we evidenced the existence of an incomplete SCO transition, with the presence of residual HS fraction at low temperature which linearly depends on the concentration of defects. Here, contrary to the previous cases, the transition temperature gradually decreases with the concentration of dopants, with a vanishing of the thermal hysteresis width due to the collapse of the elastic interactions.

The spatiotemporal aspects of these phase transitions are also investigated as functions of $R_{00}$ and p, focusing on the mode of nucleation and growth mechanism along the thermal hysteresis branches. For all studied situations of $R_{00}$, we find that the nucleation starts from the lattice corners for weak p values. However, on increasing, p, the nature of the nucleation changes significantly according to $R_{00}$ as well as on the considered branch of the thermal hysteresis. Generally, the dopant influences the lattice bond length of its SCO neighbors, which in turn acts on their spin state by creating tensile or compressive stress.

The same reasoning is developed to investigate the non-equilibrium properties of diluted SCO materials by examining the relaxation of the photo-induced metastable HS states at very low temperature. The results of the isothermal relaxation show that the temporal evolution of the HS fraction is reminiscent of that of the thermal dependence of the HS fraction. For low dopant concentrations, all relaxation curves display a sigmoidal behavior, characteristic of cooperative SCO systems. On increasing the rate of dopant, the shape of the relaxation curves changes to regular exponential or stretched exponential according to $R_{00}$ values.

In conclusion, this work demonstrates the ability of the diluted version of the electro-elastic model to mimic the thermal and the temporal behaviors of diluted SCO materials. Several possible extensions and improvements can be realized. For example, the elastic constants between the SCO and the dopant can be taken as dependent on the nature of the connected sites. In addition, dopants with sizes bigger than those of HS spin-crossover atoms are expected to lead (at low concentration) to multi-step or incomplete thermal transitions. Even more importantly, the model can be improved to include structural phase transitions coupled to the spin transition. Within this context, it is important to note that several SCO materials showing structural phase transitions were studied in the literature. One of the well documented case is that of $\left[Fe{\left(ptz\right)}_{6}{\left(BF_{4}\right)}_{2}\right]$ [19,81] (abbreviated as Fe(ptz) in the following ptz = 1-propyl-tetrazole). In this compound, the spin transition is coupled to a structural transition between an ordered high temperature structure $R\bar{3}$ and a disordered low-temperature phase. As for the pure material, Fe(ptz), the investigations of the related diluted systems show the presence of a crystallographic phase transition upon slow cooling from the HS state. In a recent study [82] of diluted $Fe_{1-x}Ru_{x}\left(ptz\right)$ single crystals, it has been found that the dilution stabilizes the LS state and induces isothermal multi-stepped relaxation, resulting from the interplay between the spin transition and the structural phase transition. These unexpected and interesting behaviors deserve to be investigated with an adapted elastic model, including lattice symmetry breaking along the spin transition [83] in order to disentangle these two contributions. Furthermore, dinuclear [84] and trinuclear SCO materials [85,86] are well known for exhibiting two- or multi-step transitions due to steric effects, causing elastic frustrations [87] between the SCO units at the transition. The control of the width of the plateau region through metal dilution is of high importance, and the extension of the present model for polynuclear SCO materials, accounting for antagonist elastic effects [33], is also a very promising objective. Finally, the recent progresses in the treatment of 3D versions [88,89] of elastic models opens new opportunities for interesting investigations of the dilution effects in more realistic lattices.

## Figures and Tables

**Table 1:** Spin states and bond length configurations used in the present model. H (L) denotes a HS (LS) site, while 0 represents the defect.

Configuration	Bond Lengths for nn	Bond Lengths for nnn
***H-H***	$R_{HH}=R\left(+1,+1\right)$	$R^{\prime }\left(+1,+1\right)=\sqrt{2}R_{HH}$
***L-L***	$R_{LL}=R\left(-1,-1\right)$	$R^{\prime }\left(-1,-1\right)=\sqrt{2}R_{LL}$
***H-L***	$R_{HL}=R\left(\pm 1,\mp 1\right)=\left(R_{HH}+R_{LL}\right)/2$	$R^{\prime }\left(\pm 1,\mp 1\right)=\sqrt{2}R_{HL}$
***0-H***	$R_{0H}=R\left(0,+1\right)=R\left(+1,\;0\right)=\frac{1}{2}\left(R_{HH}+R_{00}\right)$	$R_{0H}^{\prime }=R_{0H}\sqrt{2}$
**0-*L***	$R_{0L}=R\left(0,-1\right)=R\left(-1,\;0\right)=\frac{1}{2}\left(R_{LL}+R_{00}\right)$	$R_{0L}^{\prime }=R_{0L}\sqrt{2}$
**0-0**	$R_{00}=R\left(0,\;0\right)$	$R_{00}^{\prime }=R_{00}\sqrt{2}$

## Data Availability

Data will be made available on request.
